# Filamentous Hemagglutinin of *Bordetella pertussis* Does Not Interact with the β_2_ Integrin CD11b/CD18

**DOI:** 10.3390/ijms232012598

**Published:** 2022-10-20

**Authors:** Maryam Golshani, Waheed Ur Rahman, Adriana Osickova, Jana Holubova, Jinery Lora, Nataliya Balashova, Peter Sebo, Radim Osicka

**Affiliations:** 1 Institute of Microbiology of the Czech Academy of Sciences, Videnska 1083, 142 20 Prague, Czech Republic; 2 Department of Basic and Translational Sciences, School of Dental Medicine, University of Pennsylvania, 240 S. 40th St., Philadelphia, PA 19104, USA

**Keywords:** adenylate cyclase toxin, *Bordetella pertussis*, CD11b/CD18, filamentous hemagglutinin, heparin, integrin

## Abstract

The pertussis agent *Bordetella pertussis* produces a number of virulence factors, of which the filamentous hemagglutinin (FhaB) plays a role in *B. pertussis* adhesion to epithelial and phagocytic cells. Moreover, FhaB was recently found to play a crucial role in nasal cavity infection and *B. pertussis* transmission to new hosts. The 367 kDa FhaB protein translocates through an FhaC pore to the outer bacterial surface and is eventually processed to a ~220 kDa N-terminal FHA fragment by the SphB1 protease. A fraction of the mature FHA then remains associated with bacterial cell surface, while most of FHA is shed into the bacterial environment. Previously reported indirect evidence suggested that FHA, or its precursor FhaB, may bind the β_2_ integrin CD11b/CD18 of human macrophages. Therefore, we assessed FHA binding to various cells producing or lacking the integrin and show that purified mature FHA does not bind CD11b/CD18. Further results then revealed that the adhesion of *B. pertussis* to cells does not involve an interaction between the bacterial surface-associated FhaB and/or mature FHA and the β_2_ integrin CD11b/CD18. In contrast, FHA binding was strongly inhibited at micromolar concentrations of heparin, corroborating that the cell binding of FHA is ruled by the interaction of its heparin-binding domain with sulfated glycosaminoglycans on the cell surface.

## 1. Introduction

The Gram-negative coccobacillus *Bordetella pertussis* causes a highly contagious respiratory disease called whooping cough or pertussis. Prior to the worldwide introduction of whole-cell pertussis vaccines, some seven decades ago, pertussis used to be the leading cause of infant mortality [1,2]. Even at present, despite worldwide vaccination programs, pertussis remains the least controlled vaccine-preventable infectious disease, with ~24 million cases and more than 160,000 pertussis-related deaths worldwide each year [3].

*B. pertussis* attaches to the ciliated cells of respiratory epithelia and elicits increased mucus secretion, infiltration of inflammatory cells, and local tissue damage [1,2,4]. The bacterium is a particularly well-equipped pathogen that produces a number of virulence factors, comprising two potently immunosuppressive protein toxins, the pertussis toxin (PT) and the adenylate cyclase toxin-hemolysin (CyaA, ACT or AC-Hly), several adhesins, numerous autotransporter proteins and complement resistance factors, which play important roles in the pathogenesis of pertussis [1,2,5,6]. Among these factors, the filamentous hemagglutinin (FHA) was reported to play an important role in the adhesion to and invasion of epithelial and phagocytic cells [7,8,9,10,11,12,13,14,15,16,17]. In addition, FHA has been shown to possess immunomodulatory properties that may contribute to the impairment of host innate and adaptive immunity [17,18,19,20,21,22,23]. Due to its high immunogenicity, the mature FHA protein is included in all but the Danish acellular pertussis vaccine, although data from several studies question its protective potency as a vaccine antigen and the contribution of FHA to the effectiveness of acellular pertussis vaccines [24,25,26,27].

The FhaB protein is encoded by the structural gene *fhaB* as a 367 kDa precursor with an N-terminal signal peptide of 71 residues. Its processing leads to the formation of an N-terminal cyclic pyroglutamate, and FhaB is then exported to the surface of *B. pertussis* by a two-partner secretion system involving the FhaC outer membrane pore [28,29,30,31,32,33]. FhaB is eventually processed by bacterial proteases, primarily by the self-processing autotransporter protease SphB1, to yield the mature FHA with a size of ~220 kDa that can remain attached to the bacterial cell surface, or be released into the medium [28,29,31,34,35]. The C-terminal ~1200 residue-long ‘prodomain’ of FhaB was proposed to remain in the periplasmic space and modulate the folding of the subsequently liberated mature C-terminal domain of the processed N-terminal FHA fragment that is generated by a regulated sequential proteolytic processing of the FhaB precursor [36,37,38]. *B. pertussis* mutants lacking the SphB1 protease showed impaired mouse lung colonization capacity, suggesting that the processing and subsequent release of FHA by SphB1 may play a role in mouse lung infection [39]. However, additional roles of SphB1 in *B. pertussis* virulence were not excluded. It remains unclear whether SphB1 can inactivate some host factors involved in immune defense and/or processes, as well as other important bacterial virulence factors than FHA [17]. Moreover, processing by SphB1 appears to occur only once the extreme C-terminal segment of the prodomain of FhaB has been removed by the periplasmic protease DegP and the prodomain is subsequently cleaved by the periplasmic protease CtpA that controls the availability of SphB1 processing site in FhaB [37,38]. It has long been assumed that the processed mature form of FHA is the biologically functional form of the filamentous hemagglutinin protein, involved in bacterial adhesion. Recently, however, it has been shown that the C-terminal prodomain of the FhaB precursor of FHA plays an important role in *B. bronchiseptica* persistence in the lower respiratory tract of mice, being required for the suppression of production of the proinflammatory IL-1β cytokine and of the neutrophil and monocyte chemoattracting proteins KC (IL-8) and MCP-1, respectively [31,40,41].

The mature rod-like shaped FHA molecule, ~50 nm in length, contains four different binding domains: (i) a heparin-binding domain (HBD; residues 442 to 863) that mediates binding of FHA to sulfated proteoglycans and glycolipids, such as chondroitin sulfate and heparin, and is responsible for the hemagglutinating activity of FHA [42,43,44,45], (ii) an RGD-containing domain with an Arg-Gly-Asp (RGD) motif (residues 1097–1099), proposed to participate in FHA-dependent binding of *B. pertussis* to macrophages [9], (iii) a carbohydrate recognition domain (CRD, residues 1141 and 1279) involved in attachment of FHA to lactose-containing glycolipids of human ciliated respiratory epithelial cells and to galactose-containing glycoconjugates of macrophages [7,9,12], and (iv) a mature C-terminal domain (MCD, ~500 C-terminal residues) that was proposed to mediate *Bordetella* attachment to macrophage-like and epithelial cells and is apparently required for the colonization of rat airways by *B. bronchiseptica* and the modulation of inflammatory response to infection in mouse lungs [40].

Initially, it was proposed that the interaction of *B. pertussis* with macrophages occurs via direct binding of the RGD motif of mature FHA to the complement receptor 3 (CR3, also known as CD11b/CD18 or α_M_β_2_ integrin) [9]. However, later work showed that the RGD motif of FHA interacts with the α_v_β_3_ leukocyte response integrin (LRI, also known as CD51/CD61) and the integrin-associated CD47 protein (IAP) on human monocytes [14]. It has further been proposed that cross-linking of the LRI/IAP complex of monocytes by FHA may initiate LRI/IAP-mediated intracellular signaling via phosphatidylinositol 3-kinase (PI3K), resulting in the enhancement of CR3 binding activity for FHA, although the CR3-binding domain of FHA remains unknown [14,46]. Finally, it has been suggested that, following the initial attachment of *B. pertussis* to human respiratory epithelial cells, FHA may promote bacterial invasion of these cells via binding of the RGD motif to the very late antigen-5 (VLA-5, also known as the α_5_β_1_ integrin or CD49e/CD29) [16].

Here, we show that the mature FHA molecule purified from *B. pertussis* culture supernatants does not interact with the integrin CD11b/CD18. Moreover, we found that the adhesion of *B. pertussis* to cells does not depend on the interaction between the bacterial surface-associated FhaB/FHA and the β_2_ integrin CD11b/CD18.

## 2. Results

### 2.1. Secreted Mature FHA Does Not Bind the CD11b/CD18 Integrin

**Figure 1:**
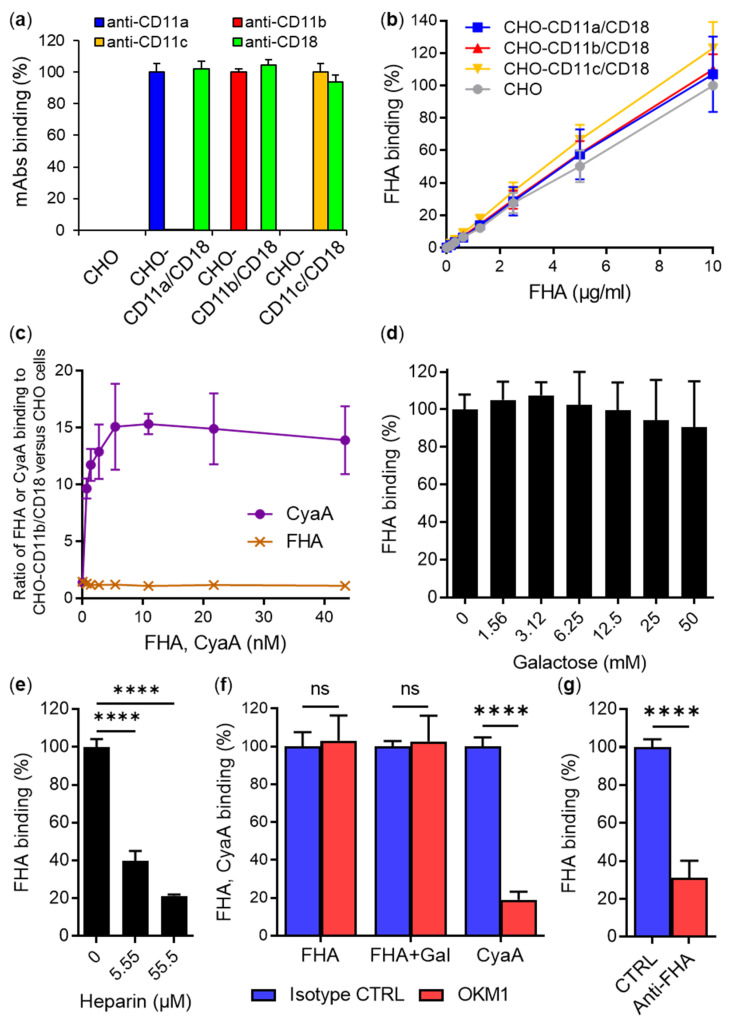
Mature FHA purified from cultures of *B. pertussis* does not recognize the CD11b/CD18 integrin. (**a**) CHO cells (2 × 10^5^) stably expressing the human integrin CD11a/CD18, CD11b/CD18, or CD11c/CD18, or mock-transfected CHO cells not expressing β_2_ integrins were incubated with anti-CD11a, anti-CD11b, anti-CD11c, and anti-CD18 mAbs for 30 min at 4 °C and analyzed by flow cytometry. Binding data were deduced from mean fluorescence intensity (MFI) values, and the mean MFI value for each mAb measured on CHO cells expressing the corresponding integrin was taken as 100%. Each bar represents the mean value with SD of three independent experiments. (**b**) CHO cells (1 × 10^5^) expressing β_2_ integrin molecules or no β_2_ integrin were incubated with different concentrations of purified and labeled FHA-Dy647 for 30 min at 4 °C and analyzed by flow cytometry. Binding data were deduced from the MFI values and expressed as a percentage of FHA binding to mock-transfected CHO cells at a concentration of 10 µg/mL (taken as 100%). Each point represents the mean value ± SD of five independent experiments. No significant differences were observed among the binding curves of FHA to different CHO variants (*p* > 0.05; ANOVA). (**c**) CD11b/CD18- or mock-transfected CHO cells (1 × 10^5^) were incubated with the indicated concentrations of purified FHA-Dy647 or CyaA-Dy647 for 30 min at 4 °C and analyzed by flow cytometry. The MFI values of FHA or CyaA bound to CHO-CD11b/CD18 cells were divided by the MFI values of FHA or CyaA bound to mock-transfected CHO cells, and the ratios were plotted as a function of protein concentration. Each point represents the mean value ± SD of five (FHA) or three (CyaA) independent experiments. While no significant differences were observed between the binding of FHA to CHO-CD11b/CD18 and mock-transfected CHO cells (*p* > 0.05; ANOVA), CyaA bound CHO-CD11b/CD18 cells with a significantly higher capacity than mock-transfected cells (*p* < 0.0001 at all tested concentrations of CyaA; ANOVA). (**d**) CHO-CD11b/CD18 cells (2 × 10^5^) were incubated with 5 µg/mL of purified FHA-Dy647 for 30 min at 4 °C in the presence of various concentrations of D-galactose (0–50 mM) and analyzed by flow cytometry. Binding data were deduced from the MFI values and expressed as a percentage of FHA binding to cells in the absence of D-galactose (taken as 100%). Each bar represents the mean value with SD of two independent experiments performed in triplicate (*p* > 0.05; ANOVA). (**e**) CHO-CD11b/CD18 cells (2 × 10^5^) were incubated with 5 µg/mL of purified FHA-Dy647 for 30 min at 4 °C in the presence of different concentrations of heparin (0, 5.55 and 55.5 µM) and analyzed by flow cytometry. Binding data were deduced from the MFI values and expressed as a percentage of FHA binding to cells in the absence of heparin (taken as 100%). Each bar represents the mean value with SD of four independent experiments performed in duplicate (****, *p* < 0.0001; ANOVA). (**f**) CHO-CD11b/CD18 cells (2 × 10^5^) were preincubated with a saturating concentration of the OKM1 mAb or an isotype control for 15 min at 4 °C and then incubated with 5 µg/mL of purified FHA-Dy647 for 30 min at 4 °C. The experiment was performed in the absence or presence of 50 mM D-galactose (Gal). In a control experiment, an equimolar concentration of CyaA-Dy647 was used instead of FHA-Dy647. After analysis of FHA and CyaA binding by flow cytometry, binding data were deduced from the MFI values and expressed as a percentage of FHA or CyaA binding to cells in the presence of the isotype control (taken as 100%). Each bar represents the mean value with SD of four (FHA) or three (CyaA) independent experiments performed in duplicate (ns, *p* > 0.05; ****, *p* < 0.0001; ANOVA). (**g**) CHO-CD11b/CD18 cells (2 × 10^5^) were incubated with 5 µg/mL of FHA-Dy647 in the presence of rabbit polyclonal anti-FHA serum or rabbit preimmune serum (CTRL) for 30 min at 4 °C and analyzed by flow cytometry. Binding data were deduced from the MFI values and expressed as a percentage of FHA binding to cells in the presence of the CTRL serum (taken as 100%). Each bar represents the mean value with SD of five independent experiments performed in duplicate (****, *p* < 0.0001; paired *t* test).

We have previously analyzed the interaction between the human β_2_ integrin CD11b/CD18 and another virulence factor of *B. pertussis*, the adenylate cyclase toxin (CyaA) [47]. Using Chinese hamster ovary (CHO) cells expressing various CD11b/CD11c chimeras in complex with CD18, we defined the segment 614–682 of the CD11b subunit as the primary binding site for CyaA on CD11b/CD18 [47]. Since the FHA of *B. pertussis* has also been reported to interact with target cells via CD11b/CD18 [9], we decided to use our CD11b constructs to also map the FHA-binding site on CD11b/CD18. However, the initial experiments revealed that purified *B. pertussis* FHA (Figure S1) does not bind the human CD11b/CD18 integrin present on the surface of transfected CHO cells. As shown in Figure 1, when exposed to 0–10 µg/mL of purified Dy647-labeled FHA at 4 °C for 30 min, the CHO cells stably expressing human CD11b/CD18 (CHO-CD11b/CD18, Figure 1a) bound similar amounts of FHA as mock-transfected CHO cells lacking CD11b/CD18 (Figure 1b). Similar results were obtained when CHO and CHO-CD11b/CD18 cells were incubated with different concentrations of purified unlabeled FHA, which was subsequently stained with an anti-FHA mAb and a secondary antibody conjugated with a fluorochrome (Figure S2). This confirmed that the Dy647-labeled FHA can be used for the binding experiments. Importantly, the binding of FHA-Dy647 to cells exhibited a linear dose–response characteristic due to the use of the directly Dy647-labeled FHA protein, which allowed for a linear detection of the bound protein amounts (Figure 1b). In contrast, a high enough concentration of the anti-FHA mAb for the detection of binding of unlabeled FHA was not available for a quantitative detection of cell-bound FHA, yielding a false saturation appearance of the binding curve, as shown in Figure S2. Therefore, Dy647-labeled FHA was further used to assess binding to CHO cells stably expressing two other β_2_ integrins, namely, CD11a/CD18 and CD11c/CD18. As shown in Figure 1b, the CHO cells expressing any of the three integrins bound similar amounts of FHA-Dy647 as mock-transfected CHO cells. In contrast, purified Dy647-labeled CyaA used at equimolar concentrations bound CHO-CD11b/CD18 cells with a substantially higher capacity than mock-transfected CHO cells over the same range of concentrations (Figure 1c). These results strongly indicate that FHA does not use CD11b/CD18, or any of the other β_2_ integrins, as a cellular receptor.

Previously, soluble D-galactose at concentrations from 5 to 50 mM was reported to reduce FHA-dependent adhesion of a PT-deficient *B. pertussis* strain (BP-TOX6) to human macrophages by ~50% [9]. From this experiment, it was deduced that FHA interacted with galactose-containing glycoconjugates on macrophages [9]. To investigate whether the binding of FHA to CHO-CD11b/CD18 cells can be inhibited by soluble D-galactose, purified Dy647-labeled FHA (5 µg/mL) was incubated with cells in the presence of a range of D-galactose concentrations (0–50 mM) for 30 min at 4 °C. As shown in Figure 1d, the binding of FHA to CHO-CD11b/CD18 cells was not inhibited, even at the highest D-galactose concentration tested. In contrast, the binding of Dy647-labeled FHA to CHO-CD11b/CD18 (Figure 1e) and CHO (Figure S3) cells was strongly inhibited in the presence of micromolar concentrations of heparin (5.55 and 55.5 µM) that is known to interact with FHA [42]. These results suggest that FHA recognizes sulfated glycans rather than galactose-containing glycoconjugates present on cell surface.

In addition, it has been previously reported that the monoclonal antibodies (mAbs) OKM1 and IB4, specific to either the CD11b or CD18 subunit of CD11b/CD18, but not mAbs recognizing other β_2_ integrins, inhibited FHA-dependent adherence of the BP-TOX6 *B. pertussis* mutant to human macrophages by ~80% in the presence of 50 mM D-galactose [9]. Based on this experiment and an experiment showing that CD18-deficient THP-1 cells exhibit negligible adhesion to surfaces coated with purified FHA, it was deduced that CD11b/CD18 serves as a receptor for *B. pertussis* by interacting directly with FHA [9]. Therefore, we performed a competition experiment with the mAb OKM1, which recognizes an epitope located between residues 614 to 682 of the CD11b subunit of CD11b/CD18 [47]. As shown in Figure 1f, CHO-CD11b/CD18 cells preincubated for 15 min at 4 °C with a saturating concentration the OKM1 mAb bound similar amounts of purified Dy647-labeled FHA as cells preincubated with an IgG2b isotype control mAb. Similarly, no inhibition of FHA binding to CHO-CD11b/CD18 cells was observed when the OKM1-blocking experiment was performed in the presence of 50 mM D-galactose (Figure 1f). In contrast to FHA, the OKM1 mAb efficiently blocked (by ~80%) the binding of an equimolar concentration of purified Dy647-labeled CyaA to CD11b/CD18 on CHO cells (Figure 1f), as previously reported [47]. In another control experiment, the binding of FHA to CHO-CD11b/CD18 cells was strongly inhibited (by ~70%) in the presence of rabbit polyclonal anti-FHA serum, but not in the presence of rabbit preimmune serum (Figure 1g). The results indicate that FHA does not compete with the specific OKM1 mAb for binding to CD11b/CD18.

**Figure 2:**
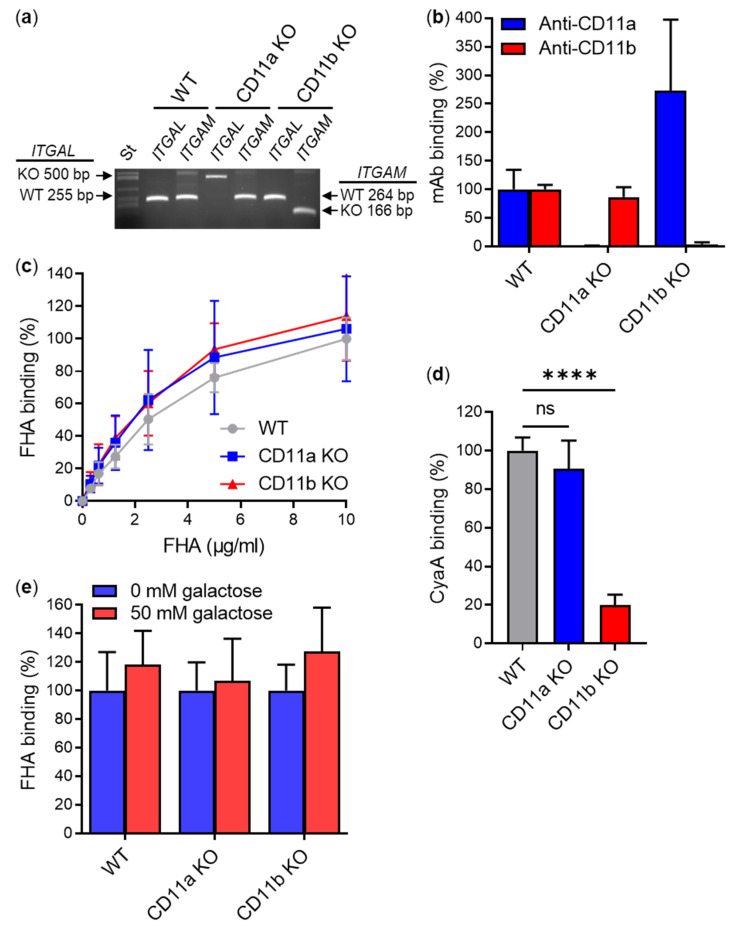
Mature FHA purified from cultures of *B. pertussis* binds CD11b KO macrophages with the same efficacy as WT macrophages. (**a**) Chromosomal DNA isolated from bone marrow-derived macrophages (1 × 10^6^) of WT, CD11a KO, and CD11b KO mice was amplified with primer pairs specific for *ITGAL* (encoding CD11a) and *ITGAM* (encoding CD11b) alleles. The product size of alleles: *ITGAL* WT, 255 bp; *ITGAM* WT 264 bp; *ITGAL* KO, 500 bp; *ITGAM* KO, 166 bp. (**b**) CD11a KO, CD11b KO and control WT mouse macrophages (1 × 10^5^) were incubated with anti-CD11a and anti-CD11b mAbs for 30 min at 4 °C and analyzed by flow cytometry. The binding of mAb to WT macrophages was taken as 100%. Each bar represents the mean value with SD of at least five independent experiments. (**c**) CD11a KO, CD11b KO and WT macrophages (1 × 10^5^) were incubated with different concentrations of purified FHA for 30 min at 4 °C, which was subsequently labeled with an anti-FHA mAb and a secondary antibody conjugated with AF488. After analysis of FHA binding by flow cytometry, binding data were deduced from the MFI values and expressed as a percentage of FHA binding to WT macrophages at a concentration of 10 µg/mL (taken as 100%). Each point represents the mean value ± SD of at least four independent experiments. No significant differences were observed among the binding curves of FHA to different variants of macrophages (*p* > 0.05; ANOVA). (**d**) CD11a KO, CD11b KO and WT macrophages (1 × 10^5^) were incubated with 5 µg/mL of purified CyaA-Dy647 for 30 min at 4 °C and analyzed by flow cytometry. Binding data were deduced from the MFI values and expressed as a percentage of CyaA binding to WT macrophages (taken as 100%). Each bar represents the mean value with SD of four independent experiments (ns, *p* > 0.05; ****, *p* < 0.0001; ANOVA). (**e**) CD11a KO, CD11b KO and WT macrophages (2 × 10^5^) were incubated with 5 µg/mL of purified FHA-Dy647 for 30 min at 4 °C in the absence or presence of 50 mM D-galactose. After analysis of FHA binding by flow cytometry, binding data were deduced from the MFI values and expressed as a percentage of FHA binding to cells in the absence of D-galactose (taken as 100%). Each bar represents the mean value with SD of five independent experiments performed in duplicate (*p* > 0.05; ANOVA).

To exclude the possibility that FHA recognizes the human CD11b/CD18 integrin expressed on macrophages, but not when expressed by transfected CHO cells, we analyzed the binding of FHA to primary mouse macrophages differentiated in the presence of macrophage colony-stimulating factor (M-CSF) from bone marrow cells (BMCs) of CD11b knockout (KO) and control (WT) C57BL/6 mice and, for comparison, also from CD11a KO mice. The presence of WT and KO alleles of the *ITGAL* (encoding CD11a) and *ITGAM* (encoding CD11b) genes in chromosomal DNA isolated from WT, CD11a KO, and CD11b KO macrophages was confirmed by genotyping (Figure 2a), and surface expression or absence of the CD11a/CD18 and CD11b/CD18 integrins was verified by flow cytometry.

As expected, an anti-CD11a mAb did not bind CD11a KO macrophages and an anti-CD11b mAb did not recognize CD11b KO macrophages, whereas both mAbs bound WT cells (Figure 2b). However, no significant differences were observed in the binding of FHA to CD11a KO, CD11b KO, and WT macrophages when cells were incubated with different amounts of purified FHA (0–10 µg/mL) at 4 °C for 30 min (Figure 2c). In contrast, CyaA, which specifically interacts with the CD11b/CD18 integrin, bound CD11a KO and WT macrophages with ~5-fold higher efficacy than the CD11b KO macrophages (Figure 2d). Further, the binding of FHA to CD11a KO, CD11b KO, and WT macrophages was not inhibited in the presence of 50 mM D-galactose (Figure 2e).

**Figure 3:**
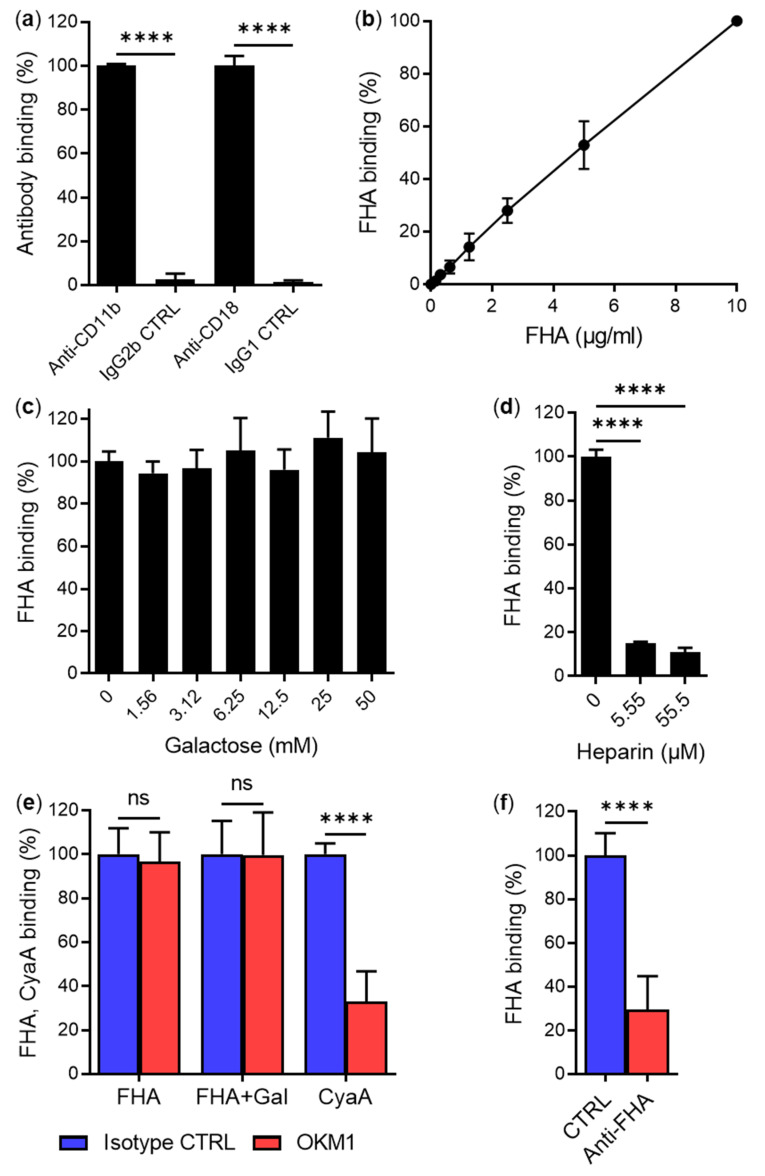
D-galactose and CD11b-specific mAb OKM1 do not reduce binding of mature FHA purified from cultures of *B. pertussis* to human macrophages. (**a**) Human macrophages (2 × 10^5^) were incubated with anti-CD11b and anti-CD18 mAbs or corresponding isotype controls for 30 min at 4 °C and analyzed by flow cytometry. The binding of mAb recognizing CD11b/CD18 on macrophages was taken as 100%. Each bar represents the mean value with SD of three independent experiments performed in duplicate (****, *p* < 0.0001; ANOVA). (**b**) Binding of mature FHA purified from cultures of *B. pertussis* to primary human macrophages. Macrophages (1 × 10^5^) were incubated with different concentrations of purified and labeled FHA-Dy647 for 30 min at 4 °C and analyzed by flow cytometry. Binding data were deduced from the MFI values and FHA binding to cells at a concentration of 10 µg/mL was taken as 100%. Each point represents the mean value ± SD of seven independent experiments. (**c**) Binding of FHA to human macrophages in the presence of D-galactose was performed as described in the legend to Figure 1d. Each bar represents the mean value with SD of three independent experiments performed in duplicate (*p* > 0.05; ANOVA). (**d**) Binding of FHA to human macrophages in the presence of heparin was performed as described in the legend to Figure 1e. Each bar represents the mean value with SD of four independent experiments performed in duplicate (****, *p* < 0.0001; ANOVA). (**e**) Binding of FHA and CyaA to human macrophages in the presence of OKM1 and D-galactose was performed as described in the legend to Figure 1f. Each bar represents the mean value with SD of five (FHA) or four (CyaA) independent experiments performed in duplicate (ns, *p* > 0.05; ****, *p* < 0.0001; ANOVA). (**f**) Binding of FHA to human macrophages in the presence of polyclonal anti-FHA serum was performed as described in the legend to Figure 1g. Each bar represents the mean value with SD of four independent experiments performed in duplicate (****, *p* < 0.0001; paired *t* test).

Since human primary macrophages were previously used in most experiments to demonstrate that FHA interacts with CD11b/CD18 and that FHA-dependent binding of *B. pertussis* to human cells can be inhibited with soluble D-galactose [9], we also used primary human monocyte-derived macrophages expressing CD11b/CD18 (Figure 3a). As shown in Figure 3b, human macrophages efficiently bound purified FHA-Dy647 without any indication of binding saturation up to 10 µg/mL at 4 °C for 30 min. To analyze whether the binding of FHA to primary macrophages is inhibited by soluble D-galactose, cells were incubated with purified Dy647-labeled FHA (5 µg/mL) in the presence of various concentrations of D-galactose ranging from 0 to 50 mM. However, as summarized in Figure 3c, no inhibition of FHA binding to primary macrophages was observed. In contrast, the binding of FHA to macrophages was efficiently blocked when heparin was used as competitor at 5.55 and 55.5 µM concentrations (Figure 3d).

Next, we tested if the CD11b-specific mAb OKM1 competes with FHA for binding to human macrophages. Cells were preincubated with a saturating concentration of OKM1 for 15 min at 4 °C and then incubated with purified Dy647-labeled FHA (5 µg/mL). However, macrophages preincubated with OKM1 bound similar amounts of FHA as macrophages preincubated with an IgG2b isotype control (Figure 3e). Similarly, no inhibition of FHA binding to macrophages was observed when the competition experiment was performed in the presence of 50 mM D-galactose (Figure 3e). On the other hand, the OKM1 mAb efficiently blocked (by ~67%) the binding of purified Dy647-labeled CyaA to primary macrophages (Figure 3e). Furthermore, the binding of FHA to macrophages was strongly inhibited (by ~70%) in the presence of rabbit polyclonal anti-FHA serum, but not in the presence of rabbit preimmune serum (Figure 3f).

All these results show that the CD11b/CD18 integrin is not specifically recognized by the mature form of FHA released by *B. pertussis* into supernatant of liquid cultures. Moreover, binding of the mature FHA molecule to different cell types (CHO cells, mouse and human macrophages) was not inhibited by high concentrations of D-galactose (50 mM) but was effectively inhibited by the four orders of magnitude lower concentration (~5 µM) of heparin.

### 2.2. FhaB/FHA Associated with the Surface of B. pertussis Cells Does Not Recognize the CD11b/CD18 Integrin

Previous experiments showing that FHA interacts with CD11b/CD18 were mostly performed with live *B. pertussis* bacteria [9]. These possess both the processed FHA bound to the bacterial cell surface and the unprocessed FhaB precursor whose exported N-terminal portion protrudes from the outer surface of the outer bacterial membrane [31]. Since it cannot be excluded that bacteria-associated mature FHA and/or FhaB may differ in interaction with CD11b/CD18 from mature FHA released by bacterial cells, we also examined the interaction between FhaB/FHA and CD11b/CD18 in the context of intact bacterial cells.

It was previously suggested that the expression of either FhaB/FHA, or of pertussis toxin (PT), is sufficient to support the adhesion of *B. pertussis* to human macrophages, because a PT-deficient strain (BP-TOX6) or an FhaB-deficient strain (BP101) bound to macrophages with similar efficacy as the parental strain (BP536), whereas a double mutant strain (BP101-TOX6) exhibited only a residual binding (6%) to macrophages [9]. The BP-TOX6 strain was thus used to show that *B. pertussis* adhesion to macrophages involves the interaction of FhaB/FHA with CD11b/CD18 [9].

**Figure 4:**
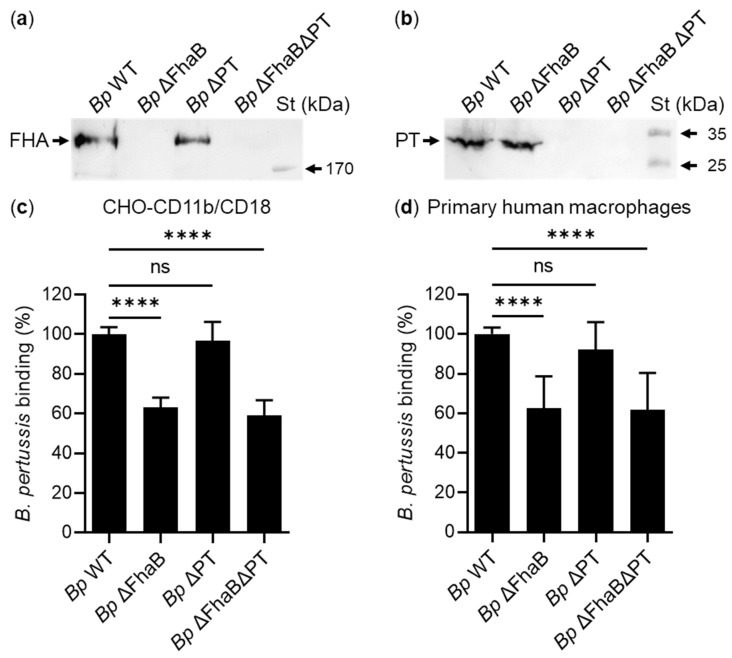
FhaB/FHA but not PT is involved in binding of the *B. pertussis* Tohama I strain to target cells. (**a**,**b**) *B. pertussis* Tohama I (*Bp* WT) and its isogenic mutants *Bp* ΔFhaB, *Bp* ΔPT, and *Bp* ΔPTΔFhaB were grown in liquid SS medium for 20 h at 37 °C and the FHA and PT proteins were detected on Western blots of bacterial lysates using anti-FHA (**a**) or anti-PT (**b**) antibodies. (**c**,**d**) CHO cells (2 × 10^5^) stably expressing the human integrin CD11b/CD18 (**c**) and primary human macrophages (2 × 10^5^) (**d**) were infected at an MOI of 100:1 with *Bp* WT or its isogenic mutant variants, all expressing mScarlet fluorescent protein, for 30 min at 37 °C. After removal of unbound bacteria, the binding of the *B. pertussis* strains to the cells was analyzed by flow cytometry. Binding data were deduced from the MFI values and expressed as a percentage of binding of each *B. pertussis* mutant strain to the parental *Bp* WT strain (taken as 100%). Each bar represents the mean value with SD of three (**c**) or five (**d**) independent experiments performed in duplicate (****, *p* < 0.0001; ns, *p* > 0.05; ANOVA).

To re-examine this observation, we constructed similar mutant strains derived from the parental *B. pertussis* Tohama I strain (*Bp* WT): (i) a mutant strain lacking expression of PT (*Bp* ΔPT); (ii) a mutant strain lacking expression of FhaB (*Bp* ΔFhaB), and (iii) a double mutant lacking expression of both virulence factors (*Bp* ΔPTΔFhaB) (Figure 4a,b). In addition, all strains were transformed with a plasmid encoding the fluorescent mScarlet protein, which allowed for direct quantification of bacterial binding to CHO-CD11b/CD18 cells and primary human macrophages by flow cytometry. Both cell types were infected with live *Bp* WT or with its isogenic mutants *Bp* ΔFhaB, *Bp* ΔPT, and *Bp* ΔPTΔFhaB at a multiplicity of infection (MOI) of 100:1 for 30 min at 37 °C. Unbound bacteria were washed away and binding of the individual *B. pertussis* strains to the cells was assessed by flow cytometry. As shown in Figure 4c,d, the *Bp* ΔFhaB and *Bp* ΔPTΔFhaB mutants bound both CHO-CD11b/CD18 cells and human macrophages significantly less efficiently (by ~40%) than the parental *Bp* WT strain. On the other hand, the FhaB/FHA-producing *Bp* ΔPT strain bound both cell types with similar efficacy as *Bp* WT (Figure 4c,d). These results show that FhaB/FHA, but not PT, is involved in the binding of the *B. pertussis* Tohama I bacteria to CHO-CD11b/CD18 cells and human macrophages.

**Figure 5:**
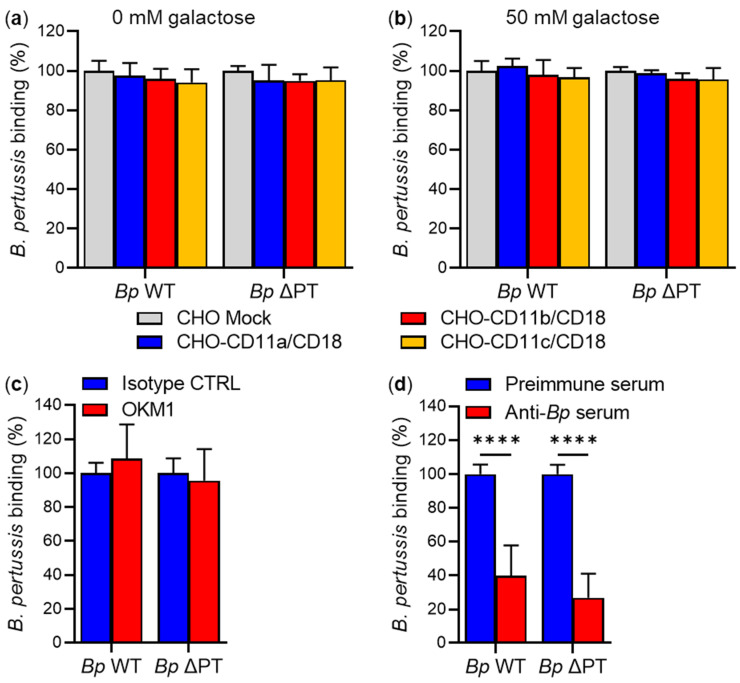
Expression of CD11b/CD18 does not facilitate adherence of *B. pertussis*. (**a**) CHO cells (2 × 10^5^) stably expressing the human integrin CD11a/CD18, CD11b/CD18, or CD11c/CD18, or mock-transfected CHO cells were infected at an MOI of 100:1 with *Bp* WT or its isogenic mutant *Bp* ΔPT, both expressing mScarlet fluorescent protein, for 30 min at 37 °C. After removal of unbound bacteria, the binding of the *B. pertussis* strains to the CHO variants was analyzed by flow cytometry. Binding data were deduced from the MFI values and expressed as a percentage of binding of each *B. pertussis* strain to mock-transfected CHO cells (taken as 100%). Each bar represents the mean value with SD of four independent experiments performed in duplicate (*p* > 0.05; ANOVA). (**b**) Binding of the *B. pertussis* strains to CHO variants in the presence of 50 mM D-galactose was performed as described in the legend to panel (**a**). Each bar represents the mean value with SD of three independent experiments performed in duplicate (*p* > 0.05; ANOVA). (**c**) CHO-CD11b/CD18 cells (2 × 10^5^) were preincubated with a saturating concentration of the mAb OKM1 or an isotype control for 15 min at RT and then infected at an MOI of 100:1 with *Bp* WT or *Bp* ΔPT for 30 min at 37 °C. After analysis of bound fluorescent bacteria by flow cytometry, binding data were deduced from the MFI values and expressed as a percentage of binding of each *B. pertussis* strain to cells in the presence of the isotype control (taken as 100%). Each bar represents the mean value with SD of five independent experiments performed in duplicate (*p* > 0.05; ANOVA). (**d**) *Bp* WT and *Bp* ΔPT were preincubated with rabbit anti-*Bp* serum or rabbit preimmune serum for 15 min at RT and then used to infect CHO-CD11b/CD18 cells (2 × 10^5^) for 30 min at 37 °C. After analysis of bound fluorescent bacteria by flow cytometry, binding data were deduced from the MFI values and expressed as a percentage of binding of each *B. pertussis* strain to cells in the presence of the preimmune serum (taken as 100%). Each bar represents the mean value with SD of five independent experiments performed in duplicate (****, *p* < 0.0001; ANOVA).

To examine whether the interaction between bacterial surface-associated FhaB/FHA and the CD11b/CD18 integrin contributes to *B. pertussis* binding to cells, we used the *Bp* WT strain and its isogenic *Bp* ΔPT mutant (an equivalent of the previously used BP-TOX6 strain [9]). First, transfected CHO cell variants expressing the three types of β_2_ integrins were infected with *Bp* WT or *Bp* ΔPT at an MOI of 100:1 for 30 min at 37 °C. Unbound bacteria were removed by the washing of cells and the binding of *Bp* WT and *Bp* ΔPT to the CHO cell variants was analyzed by flow cytometry. As summarized in Figure 5a, *Bp* WT bacteria bound the CHO-CD11b/CD18 cells with similar efficacy as the mock-transfected CHO cells, indicating that the binding of *B. pertussis* to cells was independent of the expression of the CD11b/CD18 integrin. Further, the *Bp* WT strain also bound with similar efficacy to CHO cells expressing the CD11a/CD18 and CD11c/CD18 integrins, indicating that neither of these β_2_ integrins was involved in the binding of *B. pertussis* to CHO cell surface (Figure 5a). Similar results were obtained when the adherence of the *Bp* ΔPT mutant to the CHO cell transfectants was analyzed (Figure 5a).

Similarly, no changes were observed in the binding of *Bp* WT and *Bp* ΔPT to β_2_ integrin-expressing CHO cells and to mock-transfected CHO cells in the presence of 50 mM D-galactose (Figure 5b). In addition, no inhibition of *Bp* WT or *Bp* ΔPT binding was observed upon preincubation of CHO-CD11b/CD18 cells with a saturating concentration of the CD11b-specific mAb OKM1 for 15 min at room temperature (RT) (Figure 5c). In contrast, binding of the *Bp* WT and *Bp* ΔPT strains to CHO-CD11b/CD18 cells was strongly inhibited (by ~60 and ~73%, respectively) in the presence of anti-*Bp* rabbit polyclonal serum (Figure 5d).

**Figure 6:**
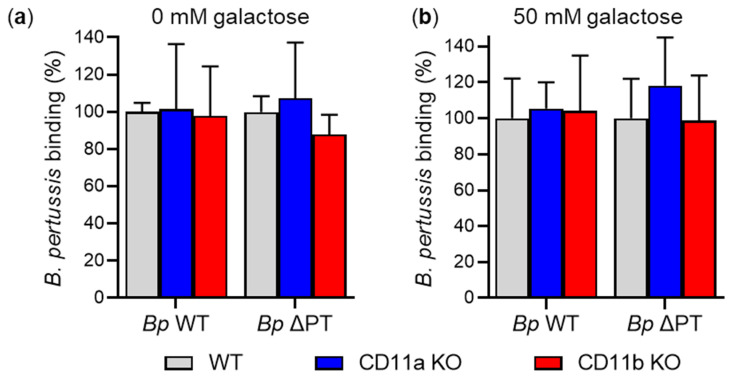
*B. pertussis* binds CD11b KO and WT macrophages with the same efficacy. (**a**) CD11a KO, CD11b KO and control WT mouse macrophages (2 × 10^5^) were infected at an MOI of 100:1 with *Bp* WT or *Bp* ΔPT expressing mScarlet fluorescent protein for 30 min at 37 °C. After removal of unbound bacteria, the binding of the *B. pertussis* strains to macrophages was analyzed by flow cytometry. Binding data were deduced from the MFI values and expressed as a percentage of binding of each *B. pertussis* strain to WT macrophages (taken as 100%). Each bar represents the mean value with SD of four independent experiments performed in duplicate (*p* > 0.05; ANOVA). (**b**) Binding of *B. pertussis* strains to macrophages in the presence of 50 mM D-galactose was performed as described in the legend to panel (**a**). Each bar represents the mean value with SD of three independent experiments performed in duplicate (*p* > 0.05; ANOVA).

Next, we analyzed the binding of *B. pertussis* strains to primary mouse macrophages. CD11a KO, CD11b KO, and WT macrophages were infected with *Bp* WT or *Bp* ΔPT at an MOI of 100:1 for 30 min at 37 °C, and the binding of bacteria to macrophages was analyzed by flow cytometry. As shown in Figure 6a, the *Bp* WT and *Bp* ΔPT bacteria bound CD11a KO, CD11b KO, and WT macrophages with comparable efficacy, showing that the binding of *B. pertussis* to macrophages does not depend on the expression of the CD11a/CD18 and CD11b/CD18 integrins. Moreover, no significant differences were observed in the binding of *B. pertussis* strains to CD11a KO, CD11b KO, and WT macrophages in the presence of 50 mM D-galactose (Figure 6b).

**Figure 7:**
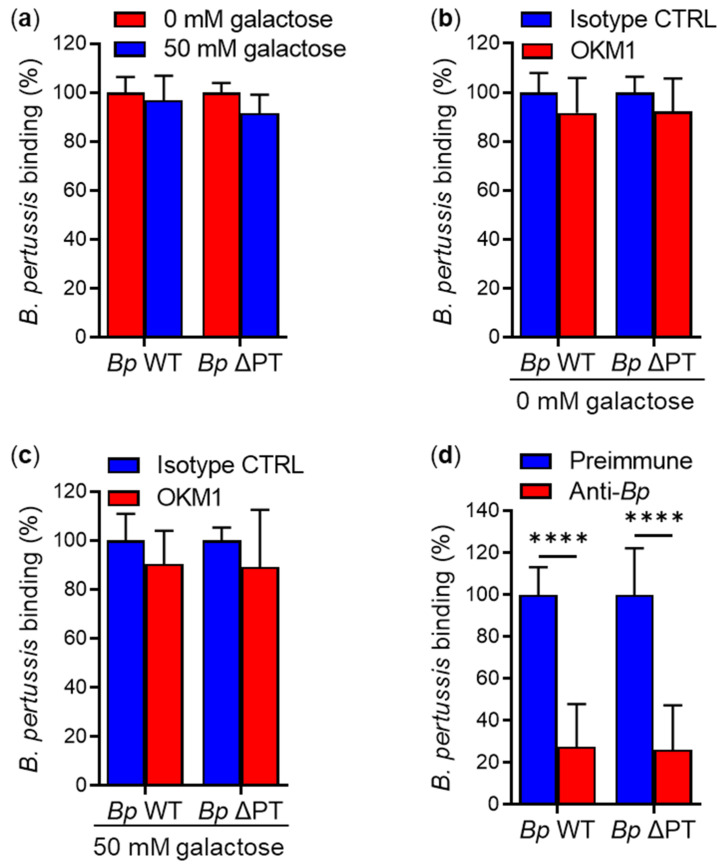
D-galactose and CD11b-specific mAb OKM1 do not reduce adherence of *B. pertussis* to human macrophages. (**a**) Primary human macrophages (2 × 10^5^) were infected at an MOI of 100:1 with mScarlet-expressing *Bp* WT or *Bp* ΔPT for 30 min at 37 °C in the absence or presence of 50 mM D-galactose. After removal of unbound bacteria, the binding of the *B. pertussis* strains to macrophages was analyzed by flow cytometry. Binding data were deduced from the MFI values and expressed as a percentage of binding of each *B. pertussis* strain to macrophages in the absence of D-galactose (taken as 100%). Each bar represents the mean value with SD of four independent experiments performed in duplicate (*p* > 0.05; ANOVA). (**b**) Binding of *B. pertussis* strains to human macrophages in the presence of the mAb OKM1 was performed as described in the legend to Figure 5c. Each bar represents the mean value with SD of five independent experiments performed in duplicate (*p* > 0.05; ANOVA). (**c**) Binding of *B. pertussis* strains to macrophages in the presence of the mAb OKM1 and 50 mM D-galactose was performed as described in the legend to Figure 5c. Each bar represents the mean value with SD of four independent experiments performed in duplicate (*p* > 0.05; ANOVA). (**d**) Binding of *B. pertussis* strains to macrophages in the presence of rabbit anti-*Bp* serum was performed as described in the legend to Figure 5d. Each bar represents the mean value with SD of four independent experiments performed in duplicate (****, *p* < 0.0001; ANOVA).

Similarly, the binding of *Bp* WT or *Bp* ΔPT to primary human macrophages was neither inhibited by 50 mM D-galactose (Figure 7a), nor by the anti-CD11b mAb OKM1 alone (Figure 7b), or in the presence of 50 mM D-galactose (Figure 7c). *Bp* WT and *Bp* ΔPT binding to macrophages was only inhibited (by ~72 and ~74%, respectively) by the rabbit anti-*Bp* serum (Figure 7d).

## 3. Discussion

Here, we have re-examined the previous reports of FhaB/FHA interaction with β_2_ integrin CD11b/CD18 and shown that the binding of *B. pertussis* to β_2_ integrin-expressing CHO cells and mouse or human macrophages does not involve any interaction between FhaB/FHA and the CD11b/CD18 integrin.

Integrins form a superfamily of 24 heterodimeric cell adhesion molecules that interact with extracellular matrix (ECM) components, soluble ligands, and cell-surface ligands and are among others involved in the regulation of vital cellular functions, such as cell proliferation, differentiation, survival, and migration [48]. Integrins have also been shown to play important roles in various host–pathogen interactions. Numerous clinically important pathogenic viruses, bacteria, and fungi express surface adhesins that can bind to integrin molecules, and several pathogens have also been shown to use integrin-mediated signaling to invade different types of host cells [49]. While only some pathogens bind integrins directly via specific adhesins, most integrin-binding pathogens interact with integrin molecules indirectly via ECM-binding adhesins. These surface molecules bind proteins of the ECM, which subsequently interact with cell surface integrins to form a molecular bridge between the pathogen and the eukaryotic cell. In this case, integrin receptors usually recognize the arginine–glycine–aspartate (RGD) motif within ECM proteins, such as vitronectin, or fibronectin [49,50].

*B. pertussis* has previously been shown to produce at least three proteins, namely FHA, FimD, and CyaA, which bind directly to integrin receptors. While the minor fimbrial subunit FimD interacts with CD49e/CD29 [51] and CyaA exhibits a high-affinity binding to the CD11b/CD18 integrin [47,52], FHA was reported to bind three different cellular integrin receptors, namely CD11b/CD18, CD51/CD61, and CD49e/CD29 [9,14,16].

Previously, the adherence of the PT-deficient *B. pertussis* strain (BP-TOX6) to human macrophages was proposed to be mediated by FHA via two separate mechanisms. One was reported to involve the RGD sequence of FHA (residues 1097–1099) recognized by the CD11b/CD18 integrin, with the other involving FHA binding to galactose-containing carbohydrates [9]. These conclusions were based on several observations. Firstly, 5 mM D-galactose reduced the FHA-dependent adhesion of BP-TOX6 bacteria to macrophages by 54%, and the adhesion was not reduced below 50% even at a 10-fold higher concentration of D-galactose. Secondly, when human macrophages were allowed to adhere to plates coated by anti-CD11b OKM1 and anti-CD18 IB4 mAbs prior to the addition of bacteria, the FHA-dependent adherence of BP-TOX6 bacteria to macrophages was reduced in the presence of 50 mM D-galactose, whereas the adhesion of BP-TOX6 bacteria to macrophages bound to anti-CD11a- or anti-CD11c-coated plastic was not affected. The authors thus concluded that sequestering of the CD11b/CD18 integrin to the antibody-coated plastic surface reduced the binding of BP-TOX6 bacteria to the apical side of macrophages. Thirdly, mutant THP-1 cells lacking CD11b/CD18 and other β_2_ integrins were found to adhere to a surface coated with purified FHA to much lower extent than parental THP-1 cells expressing the β_2_ integrins. Fourthly, the binding of BP-TOX6 bacteria to macrophages was blocked in the presence of 25 mM D-galactose over 92% with the synthetic peptide TVGRGDPHQ, comprising residues 1094–1102 of FHA, but not with a control peptide with the substitution RGD to RAD (TVGRADPHQ). Finally, a *B. pertussis* mutant derived from BP-TOX6 and expressing FHA with the substitution RGD to RAD was found to exhibit reduced binding to macrophages by 80% in the presence of 50 mM D-galactose, as compared with BP-TOX6 expressing intact FHA [9]. Importantly, all but one of these experiments were performed with the strain BP-TOX6, i.e., whole live *B. pertussis* bacteria lacking the expression of PT. It was assumed that the BP-TOX6 mutant bound human macrophages primarily via FHA, as the strain lacking both PT and FHA expression (BP101-TOX6) exhibited a strongly reduced binding capacity (~6%) to macrophages [9]. This is somewhat surprising, as BP101-TOX6 should still express several other adhesins of *B. pertussis*, such as the fimbriae or pertactin. Indeed, *B. pertussis* mutants lacking the minor (FimD) and major (Fim2 and Fim3) fimbrial subunits, or pertactin, were previously found to exhibit reduced adherence to monocytes compared to the parental W28 strain [13]. This indicates that analysis of the interaction between FHA and CD11b/CD18 in the context of live bacteria can be importantly confounded by the presence of additional *B. pertussis* adhesins.

To circumvent this difficulty, we first analyzed whether purified mature FHA binds CD11b/CD18, using stably transfected cells. This revealed that CHO cells expressing large amounts of human CD11b/CD18 on cell surface bound similar amounts of FHA as mock-transfected CHO cells not expressing CD11b/CD18 at all. Further, no difference was observed in FHA binding to CD11b KO and WT mouse macrophages. Similarly, FHA did not recognize the other β_2_ integrins, CD11a/CD18 and CD11c/CD18. Hence, purified FHA efficiently bound CHO cells or macrophages without using any β_2_ integrin as a cellular receptor. Indeed, FHA was repeatedly reported to efficiently bind to various eukaryotic cells lacking β_2_ integrins, such as ciliated respiratory cells or cultured epithelial cell lines [7,12,44,45,53]. FHA binding to these cells was found to depend on a carbohydrate recognition domain (CRD) located between residues 1141 and 1279 [12], and a heparin-binding domain (HBD) located between residues 442 to 863 [44]. The latter domain was also found to account for the hemagglutinating activity of FHA on CD11b/CD18-negative red blood cells [42,44,45]. Here, we demonstrate that micromolar concentrations of heparin, a highly sulfated glycosaminoglycan, efficiently blocked (by ~80%) FHA binding to CHO-CD11b/CD18 cells. In contrast, no significant inhibition of FHA binding to these cells was observed at up to 50 mM D-galactose concentrations. Hence, FHA primarily bound CHO cells via its heparin-binding domain, whereas the carbohydrate recognition domain was not involved in the binding of FHA to the used cells. These results are consistent with a previous report showing that heparin and other sulfated sugars (fucoidan or dextran sulfate), but not D-galactose, lactose, cellobiose, or dextran, inhibited the attachment of CHO cells to FHA-coated surface [45]. In addition, CHO cells deficient in glycosaminoglycan expression showed a substantially reduced attachment to purified FHA compared to parental CHO cells [44].

A previous study claimed that soluble D-galactose at concentrations of 5 mM to 50 mM reduced the adhesion of the BP-TOX6 strain to human macrophages by ~50%, from which it was deduced that FHA interacts with galactose-containing glycoconjugates on macrophages [9]. However, we did not observe any inhibition of FHA binding to primary human macrophages, even at 50 mM D-galactose concentration, whereas FHA binding was almost completely blocked (by ~90%) at micromolar concentrations of heparin. This indicates that purified mature FHA recognized almost exclusively sulfated glycans and not galactose-containing glycoconjugates, or the CD11b/CD18 integrin.

The previous study also reported that the FHA-dependent adherence of BP-TOX6 to macrophages was inhibited by the CD11b-specific mAb OKM1 (by ~80%) in the presence of 50 mM D-galactose [9]. However, we did not observe any significant inhibition of FHA binding to CHO-CD11b/CD18 cells or human macrophages at saturating concentrations of the OKM1 mAb in either the presence or absence of 50 mM D-galactose. These results again indicated that FHA does not directly interact with the CD11b/CD18 integrin.

In our study, we used as a control protein the purified cytotoxin CyaA of *B. pertussis*, which specifically and efficiently binds myeloid phagocytic cells via the β_2_ integrin CD11b/CD18 [52]. As expected, CyaA bound specifically and with high capacity to CHO-CD11b/CD18 cells but not to mock-transfected CHO cells, and the binding of CyaA to CHO-CD11b/CD18 cells and human macrophages was efficiently blocked by the OKM1 mAb. Moreover, CyaA bound to CD11b KO macrophages with ~5-fold lower efficacy than to WT macrophages. These control experiments with CyaA further confirmed that mature FHA does not specifically interact with the CD11b/CD18 integrin.

As described above, most of the experiments leading to the claim that FHA binds CD11b/CD18 were performed with live *B. pertussis* bacteria that harbor the unprocessed FhaB precursor on the cell surface in addition to mature FHA [9,31,34]. Therefore, we also investigated the interaction between FhaB/FHA and CD11b/CD18 in the context of live bacteria. In contrast to the previous study by Relman and colleagues [54], who visually counted fluorescein-labeled bacteria adhering to macrophages using fluorescence microscopy, we quantified the adhesion of mScarlet fluorescent protein-expressing *B. pertussis* bacteria to target cells using flow cytometry. This allowed for the rapid and accurate quantification of *B. pertussis* adhesion to ~10,000 target cells and revealed that both FhaB-deficient strains (*Bp* ΔFhaB and *Bp* ΔPTΔFhaB) were comparably impaired in binding (by ~40%) to CHO-CD11b/CD18 cells and primary human macrophages. On the other hand, the PT-deficient strain (*Bp* ΔPT) bound to both types of cells with a similar efficacy as *Bp* WT. Hence, FhaB/FHA but not PT was involved in the binding of the *B. pertussis* Tohama I bacteria to CHO cells and primary human macrophages. These results are at odds with the report of Relman and colleagues [9] that the expression of either FhaB/FHA or PT was sufficient for *B. pertussis* adhesion to human macrophages. Intriguingly, the same group reported earlier that FhaB-deficient *B. pertussis* mutant (BP101) failed to adhere to CHO cells [54]. The discrepancy between our results reported here and the observations of Relman and colleagues might possibly be due to the use of different bacterial strains for the generation of mutants (Tohama I vs. BP536) or the different method of quantification of *B. pertussis* adhesion to target cells.

On the other hand, our results are in good concordance with those of Leininger and colleagues, who showed that an FhaB-deficient *B. pertussis* 18323 mutant exhibited a significantly reduced adherence (by ~36%) to CHO cells [55]. A further decrease (by ~63%) in the adherence to CHO cells was observed with a doubly mutated *B. pertussis* 18323 deficient in FhaB and pertactin expression, suggesting a synergy of these adhesins. Similar results were observed when the adherence of the same bacterial strains to HeLa cells and P388.D1 macrophages was determined [55]. In these experiments, the adherence of [^35^S]–labeled bacteria to cells was quantified by scintillation counting [55]; hence, also using a more accurate method than is the visual counting of fluorescein-labeled bacteria by fluorescence microscopy [9].

To investigate the interaction between FhaB/FHA bound on the surface of *B. pertussis* and the CD11b/CD18 integrin, we used the *Bp* WT strain and its isogenic mutant *Bp* ΔPT (an equivalent of the previously used BP-TOX6 strain [9]). We found that *Bp* WT and *Bp* ΔPT adhered to CD11b/CD18-expressing CHO cells with a similar efficacy as to mock-transfected CHO cells. These strains also adhered with a comparable efficacy to CD11b KO and WT macrophages. Moreover, no significant changes in the adherence of *Bp* WT and *Bp* ΔPT to these cells were observed when the experiments were performed in the presence of 50 mM D-galactose. In addition, a saturating concentration of the CD11b-specific OKM1 mAb was unable to block the adherence of *Bp* WT and *Bp* ΔPT to CHO-CD11b/CD18 cells and human primary macrophages. All these results indicate that the binding of *B. pertussis* to target cells occurs independently of the interaction between the bacterial surface-associated mature FHA or its FhaB precursor and the CD11b/CD18 integrin. Hence, other FhaB/FHA-interacting counterpart(s) are involved in the binding process, such as cell surface sulfated glycosaminoglycans.

## 4. Materials and Methods

### 4.1. Antibodies

The monoclonal antibodies (mAbs) MEM-25 (mouse IgG1), BU15 (mouse IgG1), and MEM-48 (mouse IgG1) specific to human CD11a, CD11c, and CD18, respectively, and the isotype control mAbs MOPC-21 (mouse IgG1) and MPC-11 (mouse IgG2b) were obtained from Exbio, Vestec, Czech Republic. The human CD11b-specific mAb OKM1 (mouse IgG2b) was purified from the OKM1 hybridoma purchased from the European Collection of Cell Cultures, Porton Down, UK. Anti-mouse CD11a (M17/4, rat IgG2a) and anti-mouse CD11b (M1/70, rat IgG2b) mAbs were obtained from Exbio, Vestec, Czech Republic. The isotype control mAbs eBR2a (rat IgG2a) and eB149/10H5 (rat IgG2b) were from eBioscience, Thermo Fisher Scientific, Waltham, MA. All mAbs were unlabeled and/or conjugated with fluorescein isothiocyanate (FITC), Dyomics 495 (Dy495), or PerCP-eF710. Alexa Fluor 488-conjugated goat anti-mouse IgG F(ab’)_2_ fragment (GAM-AF488) was purchased from Jackson ImmunoResearch Laboratories, West Grove, PA. Horseradish peroxidase (HRP) labeled anti-mouse IgG antibody was obtained from GE Healthcare, Chicago, IL. Mouse anti-FHA F1 and F5 mAbs (kind gift of C. Locht, Institut PasteurLille, France) were isolated from previously established hybridoma lines [56]. A mouse mAb specific to the S1 subunit of PT was obtained from Santa Cruz Biotechnology, Dallas, TX. Rabbit polyclonal sera against purified FHA (anti-FHA) and whole *B. pertussis* bacteria (anti-*Bp*) were kindly provided by B. Vecerek, Institute of Microbiology, Prague, Czech Republic.

### 4.2. Bacterial Strains and Growth Conditions

*Escherichia coli* strain XL1-Blue was used for plasmid construction and protein production, and *E. coli* strain SM10 λ pir (*thi thr leu ton*A *lac*Y *sup*E *rec*A::RP4-2-Tc::Mu Km λ*pir*) was used for plasmid transfer into *B. pertussis* by bacterial conjugation. *E. coli* strains were cultured on Luria–Bertani (LB) agar medium or in LB broth at 37 °C. When appropriate, the LB culture media were supplemented with 100 μg/mL ampicillin (pSS4245 plasmid transformants).

The *B. pertussis* Tohama I strain (catalogue number CIP 81.32) was obtained from the Institute Pasteur Collection of Cultures (Paris, France). The parental and mutant *B. pertussis* strains were grown on Bordet-Gengou (BG) agar plates (Difco, USA) supplemented with 15% defibrinated sheep blood (LabMediaServis, Jaromer, Czech Republic) and 1% glycerol at 37 °C and 5% CO_2_ for ~72 h. *B. pertussis* strains containing a plasmid encoding the fluorescent mScarlet protein were grown on BG agar plates supplemented with 15 µg/mL chloramphenicol. Liquid cultures were obtained by growing *B. pertussis* bacteria in modified Stainer–Scholte (SS) medium [57] supplemented with 1 g/L heptakis (2,6-di-O-dimethyl) β-cyclodextrin and 3 g/L casamino acids.

### 4.3. Cell Lines and Growth Conditions

The CHO-K1 Chinese hamster ovary cell line (ATCC CCL-61) was obtained from the American Type Culture Collection (ATCC, Manassas, VA). CHO-K1-derived cell lines stably expressing human CD11a/CD18 (CHO-CD11a/CD18), CD11b/CD18 (CHO-CD11b/CD18), and CD11c/CD18 (CHO-CD11c/CD18) heterodimeric complexes on the cell surface or mock-transfected CHO-K1 cells (CHO) were previously prepared [47]. All CHO cell variants were grown in F12K medium (GIBCO Invitrogen, Grand Island, NY, USA) supplemented with antibiotic antimycotic solution (1000 U/mL penicillin, 0.1 mg/mL streptomycin, and 0.25 µg/mL amphotericin; Sigma-Aldrich, St. Louis, MO, USA) and 10% fetal calf serum (FCS) (GIBCO Invitrogen, Grand Island, NY, USA).

### 4.4. Primary Mouse Macrophages

Primary mouse macrophages were produced by differentiation of bone marrow cells (BMCs) in the presence of macrophage colony-stimulating factor (M-CSF), as previously described [58]. In brief, BMCs were obtained from the femurs and tibias of 8–10 week old mice, including the control strain of C57BL/6J mice (WT; JAX stock #000664), the CD11a KO mouse strain (LFA-1 KO; JAX stock #005257) [59] and the CD11b KO mouse strain (B6.129S4-Itgamtm1Myd/J; JAX stock #003991) [60]. Mice were purchased from the Jackson Laboratory, Sacramento, CA, and housed and bred at the University of Pennsylvania, Philadelphia, PA. BMCs were incubated in RPMI 1640 (Sigma-Aldrich, St. Louis, MO, USA) supplemented with 10% L929 cell-conditioned supernatant containing M-CSF, 10% FCS, and antibiotic antimycotic solution and differentiated into macrophages for 7 days at 37 °C and 5% CO_2_. The presence of WT and KO alleles of the *ITGAL* (encoding CD11a) and *ITGAM* (encoding CD11b) genes in chromosomal DNA isolated from WT, CD11a KO, and CD11b KO macrophages was verified by genotyping according to protocols provided by Jackson Laboratory, Sacramento, CA, USA.

### 4.5. Primary Human Macrophages

Primary human macrophages were produced by differentiation of monocytes isolated from buffy coats of healthy blood donors in the presence of M-CSF using a modified protocol originally published by Menck et al. [61]. In brief, 35 mL of buffy coat was layered on the top of 15 mL of Ficoll solution (1.077 g/mL) and centrifuged at 400× *g* for 30 min. The peripheral blood mononuclear cell (PBMC) layer was transferred to a new tube and washed twice with PBS supplemented with 1 mM EDTA (PBS-EDTA). For the second density gradient, 23.13 mL of Percoll solution (1.131 g/mL) was mixed with 1.87 mL of 10× concentrated PBS, and this solution was mixed with 27 mL of RPMI 1640 supplemented with 5% inactivated human AB serum (Thomayer Hospital in Prague, Czech Republic) to obtain 46% iso-osmotic Percoll solution. The PBMC solution was layered on the top of 25 mL of Percoll solution and centrifuged at 550× *g* for 30 min. The white ring of monocytes located between the two phases was collected and washed twice with PBS-EDTA. Monocytes were incubated with RPMI 1640 supplemented with 5% human AB serum, antibiotic antimycotic solution, and 50 ng/mL of human M-CSF (PeproTech EC, Ltd., London, UK) and differentiated into macrophages for 7 days at 37 °C and 5% CO_2_.

### 4.6. Construction of B. pertussis Mutant Strains

*B. pertussis* mutants were constructed by allelic exchange, as previously described [62], using the pSS4245 suicide vector (*ori*V, *Amp*R, *Str*R, *Km*R, *Ble*R, *Tet*R, and an I-*Sce*I cleavage site for counterselection) kindly provided by Dr. Scott Stibitz (U.S. CBER, FDA, Silver Spring, MD, USA). Briefly, fragments ∼700 bp in length of the upstream and downstream sequences of the open reading frames (ORFs) to be deleted were PCR amplified with appropriate primers (Table 1) and ligated into the pSS4245 vector using restriction sites in the primer tails (Table 1). The first plasmid, designated pSS4245ΔFhaB, contained an in-frame deletion of codons N4–T3588 of the *fha*B gene and was used to construct a mutant *B. pertussis* strain lacking the expression of FhaB (*Bp* ΔFhaB). The second, pSS4245ΔPT, contained an in-frame deletion of codons S1 R2–S3 C227 of the *ptx* operon and was used to construct a mutant *B. pertussis* strain lacking expression of PT (*Bp* ΔPT). To construct a double mutant *B. pertussis* strain (*Bp* ΔPTΔFhaB), the plasmid pSS4245ΔPT was introduced into the *Bp* ΔFhaB strain by allelic exchange. All deletions introduced into the chromosome of the mutant strains were verified by DNA sequencing. Finally, the plasmid pBBR1mScarlet [63] encoding the fluorescent mScarlet protein was transformed into the parental and each mutant strain.

### 4.7. Detection of FHA and PT in B. pertussis Strains by Western Blot

*B. pertussis* Tohama I and its variants lacking expression of FHA and/or PT were cultured for 20 h at 37 °C in liquid SS medium, and cells from 1 mL of the bacterial culture were collected by centrifugation (10 min, 15,000× *g*). The pelleted cells were lyzed in 100 µL of TUS buffer (50 mM Tris-HCl (pH 8.0), 8 M urea, 2% (*w*/*v*) SDS), mixed with 50 µL of Laemmli buffer (50 mM Tris-HCl (pH 6.8), 2% (*w*/*v*) SDS, 10% glycerol (*v*/*v*), 0.1% (*w*/*v*) bromophenol blue, 1% (*v*/*v*) β-mercaptoethanol) and heated at 100 °C for 5 min. Whole bacterial cell lysates were separated by SDS-PAGE and transferred to a nitrocellulose membrane (Pall Corporation, Pensacola, FL). The membrane was blocked with 5% BSA in PBS for 2 h at RT and incubated with a mouse mAb specific to the S1 subunit of PT (diluted 1:500) or with mouse anti-FHA F1 mAb (diluted 1:100) for 1 h at RT. After washing with PBS, the membrane was incubated with HRP-conjugated anti-mouse IgG antibody (diluted 1:5000) for 1 h at RT and washed with PBS. Blots were developed using SuperSignal West Femto maximum sensitivity substrate (Thermo Scientific, Rockford, IL, USA) and the chemiluminescent signal was recorded using an ImageQuant LAS 4000 Imager (GE Healthcare Bio-Sciences AB, Uppsala, Sweden).

### 4.8. Production, Purification and Labeling of Mature FHA

Mature FHA was purified by affinity chromatography from the supernatant of a *B. pertussis* culture grown at 37 °C in modified SS medium to an OD_650 nm_ ≥ 4.0. The bacterial culture (1 L) was centrifuged at 10,000× *g* for 20 min at 4 °C, and the supernatant was filtered through a 0.44 µm pore filter. The bacteria-free supernatant was loaded onto an Econo-Pac 10 mL disposable chromatography column (Bio-Rad, Prague, Czech Republic) packed with 5 mL of a Cellufine sulfate gel slurry (JNC Corporation, Tokyo, Japan) and equilibrated with 10 mM sodium phosphate buffer (pH 7.6). After washing the column with 50 mL of equilibration buffer, FHA was eluted with equilibration buffer containing 1 M NaCl.

To label FHA with Dyomics 647 (Dy647), purified FHA was mixed with Dy647-NHS ester (Dyomics, Jena, Germany) at a concentration that gave a molar ratio of Dy647:FHA of ~5:1. Labeling was performed at RT for 2 h, and the labeled FHA protein (FHA-Dy647) was dialyzed overnight at 4 °C against PBS buffer.

### 4.9. Production, Purification and Labeling of CyaA

CyaC-acylated CyaA was expressed from the pT7CACT1 plasmid [64] in the *E. coli* strain XL1-Blue (Stratagene, La Jolla, CA). CyaA was purified by a combination of ion exchange chromatography on DEAE-Sepharose CL-6B (Sigma–Aldrich, St. Louis, MO) and hydrophobic chromatography on Phenyl-Sepharose CL-4B (Sigma–Aldrich, St. Louis, MO, USA), as previously described [64]. Labeling of CyaA with Dy647-NHS ester (Dyomics, Jena, Germany) was performed on the Phenyl-Sepharose CL-4B column after the DEAE-Sepharose purification step, as previously described [47].

### 4.10. Binding of FHA, CyaA, and mAbs to Cells

For FHA or CyaA binding assays, CHO cell variants or macrophages were incubated in 100 µL of cHBSS buffer (10 mM HEPES, pH 7.4, 140 mM NaCl, 5 mM KCl, 2 mM CaCl_2_, 2 mM MgCl_2_, 1% (*v*/*v*) FCS, 1% (*w*/*v*) glucose) containing the indicated concentrations of FHA-Dy647 or CyaA-Dy647 for 30 min at 4 °C. In some experiments, cells were incubated with unlabeled FHA, and surface-bound FHA was stained with primary mouse anti-FHA F5 mAb (diluted 1:100) and secondary GAM-AF488 mAb (diluted 1:200).

For staining of β_2_ integrins on the cell surface with mAbs, cells were incubated for 30 min at 4 °C in 50 µL of cHBSS buffer containing mAbs diluted according to the manufacturer’s instructions.

After washing, cells were resuspended in cHBSS and analyzed by flow cytometry in the presence of 1 µg/mL Hoechst 33258. Data were analyzed using FlowJo software (Tree Star, Ashland, OR, USA) and appropriate gating was used to exclude cell aggregates and dead cells.

### 4.11. Binding of FHA to Cells in the Presence of D-Galactose, Heparin, OKM1 mAb, and Anti-FHA Serum

For the binding of FHA to cells in the presence of D-galactose or heparin, 5 µg/mL of FHA-Dy647 was preincubated with various concentrations of D-galactose (0–50 mM) or heparin (5.55 and 55.5 µM) in HBSS-Ca/Mg buffer (10 mM HEPES (pH 7.4), 140 mM NaCl, 5 mM KCl, 2 mM CaCl_2_, 2 mM MgCl_2_, 1% (*v*/*v*) FCS) for 15 min at 4 °C. Pellets of 2 × 10^5^ CHO-CD11b/CD18 cells or macrophages were then resuspended in 100 µL of each mixture and incubated for 30 min at 4 °C. After washing with HBSS-Ca/Mg buffer, the binding of FHA to cells was determined by flow cytometry as described above.

For the binding of FHA to CD11b/CD18 in the presence of the OKM1 mAb, 2 × 10^5^ CHO-CD11b/CD18 cells or macrophages were preincubated in the presence of saturating concentrations of the OKM1 mAb (25 µg/mL) in 45 µL of HBSS-Ca/Mg buffer for 15 min at 4 °C. Cells preincubated with an isotype control mAb (25 µg/mL) served as controls. Subsequently, 5 µL of purified FHA-Dy647 was added to the cells to a final concentration of 5 µg/mL, and incubation was continued at 4 °C for 30 min. After washing with HBSS-Ca/Mg buffer, the binding of FHA to cells was determined by flow cytometry. When the OKM1-blocking experiment was performed in the presence of D-galactose, HBSS-Ca/Mg buffer supplemented with 50 mM galactose was used instead of HBSS-Ca/Mg. In a control experiment, an equimolar concentration of CyaA-Dy647 was used instead of FHA-Dy647.

For the binding of FHA to cells in the presence of anti-FHA serum, 5 µg/mL of purified FHA-Dy647 was preincubated with polyclonal rabbit anti-FHA serum (diluted 1:10) or rabbit preimmune serum (diluted 1:10) in cHBSS buffer for 15 min at 4 °C. Pellets of 2 × 10^5^ CHO-CD11b/CD18 cells or macrophages were then resuspended in 50 µL of each mixture and incubated for 30 min at 4 °C. After washing with cHBSS, the binding of FHA to cells was determined by flow cytometry, as described above.

### 4.12. Binding of B. pertussis Strains to Cells

Pellets of 2 × 10^5^ CHO cell variants or macrophages were resuspended in 100 µL of RPMI 1640 containing 2 × 10^7^ mScarlet-expressing *B. pertussis* strains (*Bp* WT, *Bp* ΔFhaB, *Bp* ΔPT, and *Bp* ΔPTΔFhaB) to achieve an MOI of 100. After centrifugation (640× *g* for 5 min at RT), the cells were incubated with the bacterial strains for 30 min at 37 °C. After the removal of unbound bacteria by washing with PBS, the samples were fixed with 4% paraformaldehyde (PFA) for 15 min at RT and the binding of the *B. pertussis* strains to cells was analyzed by flow cytometry.

### 4.13. Binding of B. pertussis Strains to Cells in the Presence of D-Galactose, OKM1 mAb, and Anti-B. pertussis Serum

For the binding of *B. pertussis* to cells in the presence of D-galactose, 2 × 10^7^ mScarlet-expressing *B. pertussis* strains were preincubated with 50 mM D-galactose in 100 µL of RPMI 1640 for 15 min at RT and then added to pellets of 2 × 10^5^ CHO cell variants or macrophages.

For the binding of *B. pertussis* to CD11b/CD18 in the presence of the OKM1 mAb, 2 × 10^5^ CHO-CD11b/CD18 cells or macrophages were preincubated in the presence of saturating concentrations of the OKM1 mAb (25 µg/mL) or an isotype control mAb (25 µg/mL) in 45 µL of RPMI 1640 for 15 min at RT. The mAb-preincubated cells were then infected by adding 5 µL of 2 × 10^7^ mScarlet-expressing *B. pertussis* strains to reach a final MOI of 100. Binding of the *B. pertussis* strains to mAb-preincubated human macrophages was also performed in the presence of 50 mM D-galactose.

For the binding of *B. pertussis* to cells in the presence of anti-*Bp* serum, 2 × 10^7^ mScarlet-expressing *B. pertussis* strains were preincubated with polyclonal rabbit anti-*Bp* serum (diluted 1:10) or preimmune rabbit serum (diluted 1:10) in 5 µL of RPMI 1640 for 15 min at RT. The mixtures were then used to infect 2 × 10^5^ CHO-CD11b/CD18 cells or human macrophages resuspended in 45 µL of RPMI 1640.

After centrifugation (640× *g* for 5 min at RT), the cells were incubated with the bacterial strains in the presence of D-galactose, mAbs or rabbit sera for 30 min at 37 °C. After removal of unbound bacteria by washing with PBS, samples were fixed with 4% PFA for 15 min at RT and the binding of the *B. pertussis* strains to cells was analyzed by flow cytometry.

### 4.14. Ethical Statement

Commercial anonymous human buffy coats were obtained from the Blood Bank of Thomayer Hospital, Prague, Czech Republic, so informed consent was not required. Human cells were handled in accordance with the safety and quality requirements of Act No. 296/2008 Coll. and Decree No. 422/2008 Coll., and the protocols used were in accordance with the internal guidelines of the Institute of Microbiology of the CAS, v. v. i. All experiments performed with animals were approved by the Animal Welfare Committee at the University of Pennsylvania under the Animal Welfare Assurance Number A3079-01 and Animal protocol number 806760.

### 4.15. Statistical Analysis

Results were expressed as the arithmetic mean ± standard deviation (SD) of the mean. Statistical analysis was performed using Student’s *t-*test, one-way ANOVA followed by Dunnett’s post-test, or two-way ANOVA with Sidak’s multiple comparisons test. GraphPad Prism 9.1.0 (GraphPad Software, La Jolla, CA, USA) was used to perform statistical analysis. Significant differences are indicated by asterisks (*, *p* < 0.05; **, *p* < 0.01; ***, *p* < 0.001; ****, *p* < 0.0001).

## Figures and Tables

**Table 1:** Nucleotide sequences of the primers used to construct *Bp* ΔPT and *Bp* ΔFhaB.

Strain	Primer Name	Primer Sequence ^1^
*Bp* ΔPT	ΔPtx **SpeI** for	CT**ACTAGT**GCGGTGCTGGAACATATCC
	ΔPtx **SacI** rev	CT**GAGCTC**CATCCCGTCTTCCCCTCTG
	ΔPtx **SacI** for	CT**GAGCTC**TGAGCCGCCGGCTCGGATC
	ΔPtx **BamHI** rev	CT**GGATCC**CAGCGGCGCATAGACGGTAC
*Bp* ΔFHA	ΔFhaB **NotI** for	CT**GCGGCCGC**GGCATTGATGACCTCGTGCAG
	ΔFhaB **SpeI** rev	GA**ACTAGT**CGTGTTCATATTCCGACCAGC
	ΔFhaB **SpeI** for	CT**ACTAGT**AACAAATAGGTAGTCGCGGCCTG
	ΔFhaB **BamHI**rev	GA**GGATCC**CATGCCGCCTTGCCGCTTTAC

^1^ Restriction sites and enzymes used for cloning of PCR fragments into the pSS4245 vector are shown in bold and underlined.

## Supplementary Materials

The following supporting information can be downloaded at: https://www.mdpi.com/article/10.3390/ijms232012598/s1.

## References

[1] Mattoo S., Cherry J.D. (2005). Molecular pathogenesis, epidemiology, and clinical manifestations of respiratory infections due to *Bordetella pertussis* and other *Bordetella* subspecies. Clin. Microbiol. Rev..

[2] Melvin J.A., Scheller E.V., Miller J.F., Cotter P.A. (2014). *Bordetella pertussis* pathogenesis: Current and future challenges. Nat. Rev. Microbiol..

[3] Yeung K.H.T., Duclos P., Nelson E.A.S., Hutubessy R.C.W. (2017). An update of the global burden of pertussis in children younger than 5 years: A modelling study. Lancet Infect. Dis..

[4] Malandra A., Rahman W.U., Klimova N., Streparola G., Holubova J., Osickova A., Bariselli S., Sebo P., Osicka R. (2021). *Bordetella* Adenylate Cyclase Toxin Elicits Airway Mucin Secretion through Activation of the cAMP Response Element Binding Protein. Int. J. Mol. Sci..

[5] Novak J., Cerny O., Osickova A., Linhartova I., Masin J., Bumba L., Sebo P., Osicka R. (2017). Structure-Function Relationships Underlying the Capacity of *Bordetella* Adenylate Cyclase Toxin to Disarm Host Phagocytes. Toxins.

[6] Masin J., Osicka R., Bumba L., Sebo P. (2015). *Bordetella* adenylate cyclase toxin: A unique combination of a pore-forming moiety with a cell-invading adenylate cyclase enzyme. Pathog. Dis..

[7] Tuomanen E., Towbin H., Rosenfelder G., Braun D., Larson G., Hansson G.C., Hill R. (1988). Receptor analogs and monoclonal antibodies that inhibit adherence of *Bordetella pertussis* to human ciliated respiratory epithelial cells. J. Exp. Med..

[8] Ewanowich C.A., Melton A.R., Weiss A.A., Sherburne R.K., Peppler M.S. (1989). Invasion of HeLa 229 cells by virulent *Bordetella pertussis*. Infect. Immun..

[9] Relman D., Tuomanen E., Falkow S., Golenbock D.T., Saukkonen K., Wright S.D. (1990). Recognition of a bacterial adhesion by an integrin: Macrophage CR3 (alpha M beta 2, CD11b/CD18) binds filamentous hemagglutinin of *Bordetella pertussis*. Cell.

[10] Saukkonen K., Cabellos C., Burroughs M., Prasad S., Tuomanen E. (1991). Integrin-mediated localization of *Bordetella pertussis* within macrophages: Role in pulmonary colonization. J. Exp. Med..

[11] Friedman R.L., Nordensson K., Wilson L., Akporiaye E.T., Yocum D.E. (1992). Uptake and intracellular survival of *Bordetella pertussis* in human macrophages. Infect. Immun..

[12] Prasad S.M., Yin Y., Rodzinski E., Tuomanen E.I., Masure H.R. (1993). Identification of a carbohydrate recognition domain in filamentous hemagglutinin from *Bordetella pertussis*. Infect. Immun..

[13] Hazenbos W.L., van den Berg B.M., van′t Wout J.W., Mooi F.R., van Furth R. (1994). Virulence factors determine attachment and ingestion of nonopsonized and opsonized *Bordetella pertussis* by human monocytes. Infect. Immun..

[14] Ishibashi Y., Claus S., Relman D.A. (1994). *Bordetella pertussis* filamentous hemagglutinin interacts with a leukocyte signal transduction complex and stimulates bacterial adherence to monocyte CR3 (CD11b/CD18). J. Exp. Med..

[15] Bassinet L., Gueirard P., Maitre B., Housset B., Gounon P., Guiso N. (2000). Role of adhesins and toxins in invasion of human tracheal epithelial cells by *Bordetella pertussis*. Infect. Immun..

[16] Ishibashi Y., Relman D.A., Nishikawa A. (2001). Invasion of human respiratory epithelial cells by *Bordetella pertussis*: Possible role for a filamentous hemagglutinin Arg-Gly-Asp sequence and alpha5beta1 integrin. Microb. Pathog..

[17] Villarino Romero R., Osicka R., Sebo P. (2014). Filamentous hemagglutinin of *Bordetella pertussis*: A key adhesin with immunomodulatory properties?. Future Microbiol..

[18] Abramson T., Kedem H., Relman D.A. (2001). Proinflammatory and proapoptotic activities associated with *Bordetella pertussis* filamentous hemagglutinin. Infect. Immun..

[19] Abramson T., Kedem H., Relman D.A. (2008). Modulation of the NF-kappaB pathway by *Bordetella pertussis* filamentous hemagglutinin. PLoS ONE.

[20] Dieterich C., Relman D.A. (2011). Modulation of the host interferon response and ISGylation pathway by *B. pertussis* filamentous hemagglutinin. PLoS ONE.

[21] Henderson M.W., Inatsuka C.S., Sheets A.J., Williams C.L., Benaron D.J., Donato G.M., Gray M.C., Hewlett E.L., Cotter P.A. (2012). Contribution of *Bordetella* filamentous hemagglutinin and adenylate cyclase toxin to suppression and evasion of interleukin-17-mediated inflammation. Infect. Immun..

[22] McGuirk P., Mills K.H. (2000). Direct anti-inflammatory effect of a bacterial virulence factor: IL-10-dependent suppression of IL-12 production by filamentous hemagglutinin from *Bordetella pertussis*. Eur. J. Immunol..

[23] McGuirk P., McCann C., Mills K.H. (2002). Pathogen-specific T regulatory 1 cells induced in the respiratory tract by a bacterial molecule that stimulates interleukin 10 production by dendritic cells: A novel strategy for evasion of protective T helper type 1 responses by *Bordetella pertussis*. J. Exp. Med..

[24] Munoz J.J., Arai H., Cole R.L. (1981). Mouse-protecting and histamine-sensitizing activities of pertussigen and fimbrial hemagglutinin from *Bordetella pertussis*. Infect. Immun..

[25] Cherry J.D., Gornbein J., Heininger U., Stehr K. (1998). A search for serologic correlates of immunity to *Bordetella pertussis* cough illnesses. Vaccine.

[26] Watanabe M., Nakase Y. (1982). Mutant of *Bordetella pertussis* which lacks ability to produce filamentous hemagglutinin. Infect. Immun..

[27] Poolman J.T. (2014). Shortcomings of pertussis vaccines: Why we need a third generation vaccine. Expert Rev. Vaccines.

[28] Delisse-Gathoye A.M., Locht C., Jacob F., Raaschou-Nielsen M., Heron I., Ruelle J.L., de Wilde M., Cabezon T. (1990). Cloning, partial sequence, expression, and antigenic analysis of the filamentous hemagglutinin gene of *Bordetella pertussis*. Infect. Immun..

[29] Domenighini M., Relman D., Capiau C., Falkow S., Prugnola A., Scarlato V., Rappuoli R. (1990). Genetic characterization of *Bordetella pertussis* filamentous haemagglutinin: A protein processed from an unusually large precursor. Mol. Microbiol..

[30] Lambert-Buisine C., Willery E., Locht C., Jacob-Dubuisson F. (1998). N-terminal characterization of the *Bordetella pertussis* filamentous haemagglutinin. Mol. Microbiol..

[31] Mazar J., Cotter P.A. (2006). Topology and maturation of filamentous haemagglutinin suggest a new model for two-partner secretion. Mol. Microbiol..

[32] Maier T., Clantin B., Gruss F., Dewitte F., Delattre A.S., Jacob-Dubuisson F., Hiller S., Villeret V. (2015). Conserved Omp85 lid-lock structure and substrate recognition in FhaC. Nat. Commun..

[33] Guerin J., Bigot S., Schneider R., Buchanan S.K., Jacob-Dubuisson F. (2017). Two-Partner Secretion: Combining Efficiency and Simplicity in the Secretion of Large Proteins for Bacteria-Host and Bacteria-Bacteria Interactions. Front. Cell. Infect. Microbiol..

[34] Coutte L., Antoine R., Drobecq H., Locht C., Jacob-Dubuisson F. (2001). Subtilisin-like autotransporter serves as maturation protease in a bacterial secretion pathway. EMBO J..

[35] Jurnecka D., Man P., Sebo P., Bumba L. (2018). *Bordetella pertussis* and *Bordetella bronchiseptica* filamentous hemagglutinins are processed at different sites. FEBS Open Bio..

[36] Noel C.R., Mazar J., Melvin J.A., Sexton J.A., Cotter P.A. (2012). The prodomain of the *Bordetella* two-partner secretion pathway protein FhaB remains intracellular yet affects the conformation of the mature C-terminal domain. Mol. Microbiol..

[37] Nash Z.M., Cotter P.A. (2019). Regulated, sequential processing by multiple proteases is required for proper maturation and release of *Bordetella* filamentous hemagglutinin. Mol. Microbiol..

[38] Johnson R.M., Nash Z.M., Dedloff M.R., Shook J.C., Cotter P.A. (2021). DegP Initiates Regulated Processing of Filamentous Hemagglutinin in *Bordetella bronchiseptica*. mBio.

[39] Coutte L., Alonso S., Reveneau N., Willery E., Quatannens B., Locht C., Jacob-Dubuisson F. (2003). Role of adhesin release for mucosal colonization by a bacterial pathogen. J. Exp. Med..

[40] Julio S.M., Inatsuka C.S., Mazar J., Dieterich C., Relman D.A., Cotter P.A. (2009). Natural-host animal models indicate functional interchangeability between the filamentous haemagglutinins of *Bordetella pertussis* and *Bordetella bronchiseptica* and reveal a role for the mature C-terminal domain, but not the RGD motif, during infection. Mol. Microbiol..

[41] Melvin J.A., Scheller E.V., Noel C.R., Cotter P.A. (2015). New Insight into Filamentous Hemagglutinin Secretion Reveals a Role for Full-Length FhaB in *Bordetella* Virulence. mBio.

[42] Menozzi F.D., Gantiez C., Locht C. (1991). Interaction of the *Bordetella pertussis* filamentous hemagglutinin with heparin. FEMS Microbiol. Lett..

[43] Makhov A.M., Hannah J.H., Brennan M.J., Trus B.L., Kocsis E., Conway J.F., Wingfield P.T., Simon M.N., Steven A.C. (1994). Filamentous hemagglutinin of *Bordetella pertussis*. A bacterial adhesin formed as a 50-nm monomeric rigid rod based on a 19-residue repeat motif rich in beta strands and turns. J. Mol. Biol..

[44] Hannah J.H., Menozzi F.D., Renauld G., Locht C., Brennan M.J. (1994). Sulfated glycoconjugate receptors for the *Bordetella pertussis* adhesin filamentous hemagglutinin (FHA) and mapping of the heparin-binding domain on FHA. Infect. Immun..

[45] Menozzi F.D., Mutombo R., Renauld G., Gantiez C., Hannah J.H., Leininger E., Brennan M.J., Locht C. (1994). Heparin-inhibitable lectin activity of the filamentous hemagglutinin adhesin of *Bordetella pertussis*. Infect. Immun..

[46] Ishibashi Y., Yoshimura K., Nishikawa A., Claus S., Laudanna C., Relman D.A. (2002). Role of phosphatidylinositol 3-kinase in the binding of *Bordetella pertussis* to human monocytes. Cell. Microbiol..

[47] Osicka R., Osickova A., Hasan S., Bumba L., Cerny J., Sebo P. (2015). *Bordetella* adenylate cyclase toxin is a unique ligand of the integrin complement receptor 3. eLife.

[48] Arnaout M.A., Mahalingam B., Xiong J.P. (2005). Integrin structure, allostery, and bidirectional signaling. Annu. Rev. Cell Dev. Biol..

[49] Ulanova M., Gravelle S., Barnes R. (2009). The role of epithelial integrin receptors in recognition of pulmonary pathogens. J. Innate Immun..

[50] Ruoslahti E. (1996). RGD and other recognition sequences for integrins. Annu. Rev. Cell Dev. Biol..

[51] Hazenbos W.L., van den Berg B.M., Geuijen C.W., Mooi F.R., van Furth R. (1995). Binding of FimD on *Bordetella pertussis* to very late antigen-5 on monocytes activates complement receptor type 3 via protein tyrosine kinases. J. Immunol..

[52] Guermonprez P., Khelef N., Blouin E., Rieu P., Ricciardi-Castagnoli P., Guiso N., Ladant D., Leclerc C. (2001). The adenylate cyclase toxin of *Bordetella pertussis* binds to target cells via the alpha(M)beta(2) integrin (CD11b/CD18). J. Exp. Med..

[53] Leininger E., Ewanowich C.A., Bhargava A., Peppler M.S., Kenimer J.G., Brennan M.J. (1992). Comparative roles of the Arg-Gly-Asp sequence present in the *Bordetella pertussis* adhesins pertactin and filamentous hemagglutinin. Infect. Immun..

[54] Relman D.A., Domenighini M., Tuomanen E., Rappuoli R., Falkow S. (1989). Filamentous hemagglutinin of *Bordetella pertussis*: Nucleotide sequence and crucial role in adherence. Proc. Natl. Acad. Sci. USA.

[55] Leininger E., Kenimer J.G., Brennan M.J., Manclark C.R. (1990). Surface Proteins of *Bordetella pertussis*: Role in Adherence. Proceedings of the Sixth International Symposium on Pertussis.

[56] Irons L.I., Ashworth L.A., Wilton-Smith P. (1983). Heterogeneity of the filamentous haemagglutinin of *Bordetella pertussis* studied with monoclonal antibodies. J. Gen. Microbiol..

[57] Stainer D.W., Scholte M.J. (1970). A simple chemically defined medium for the production of phase I *Bordetella pertussis*. J. Gen. Microbiol..

[58] Rahman W.U., Osickova A., Klimova N., Lora J., Balashova N., Osicka R. (2020). Binding of *Kingella kingae* RtxA Toxin Depends on Cell Surface Oligosaccharides, but Not on beta2 Integrins. Int. J. Mol. Sci..

[59] Ding Z.M., Babensee J.E., Simon S.I., Lu H., Perrard J.L., Bullard D.C., Dai X.Y., Bromley S.K., Dustin M.L., Entman M.L. (1999). Relative contribution of LFA-1 and Mac-1 to neutrophil adhesion and migration. J. Immunol..

[60] Coxon A., Rieu P., Barkalow F.J., Askari S., Sharpe A.H., von Andrian U.H., Arnaout M.A., Mayadas T.N. (1996). A novel role for the beta 2 integrin CD11b/CD18 in neutrophil apoptosis: A homeostatic mechanism in inflammation. Immunity.

[61] Menck K., Behme D., Pantke M., Reiling N., Binder C., Pukrop T., Klemm F. (2014). Isolation of human monocytes by double gradient centrifugation and their differentiation to macrophages in teflon-coated cell culture bags. J. Vis. Exp. JoVE.

[62] Inatsuka C.S., Xu Q., Vujkovic-Cvijin I., Wong S., Stibitz S., Miller J.F., Cotter P.A. (2010). Pertactin is required for *Bordetella* species to resist neutrophil-mediated clearance. Infect. Immun..

[63] Klimova N., Holubova J., Streparola G., Tomala J., Brazdilova L., Stanek O., Bumba L., Sebo P. (2022). Pertussis toxin suppresses dendritic cell-mediated delivery of *B. pertussis* into lung-draining lymph nodes. PLoS Pathog..

[64] Osicka R., Osickova A., Basar T., Guermonprez P., Rojas M., Leclerc C., Sebo P. (2000). Delivery of CD8(+) T-cell epitopes into major histocompatibility complex class I antigen presentation pathway by *Bordetella pertussis* adenylate cyclase: Delineation of cell invasive structures and permissive insertion sites. Infect. Immun..

