# Supplementary material for: Filamentous Hemagglutinin of Bordetella pertussis Does Not Interact with the β2 Integrin CD11b/CD18
Source: Int J Mol Sci. 2022 Oct 20;23(20):12598. doi: 10.3390/ijms232012598 (PMC9604300; doi:10.3390/ijms232012598)
Supplement: Supplementary file 1 [file ijms-23-12598-s001.zip › ijms-1918354-supplementary.pdf]

Supplementary Materials:

Filamentous hemagglutinin of *Bordetella pertussis* does not interact with the β2 integrin CD11b/CD18

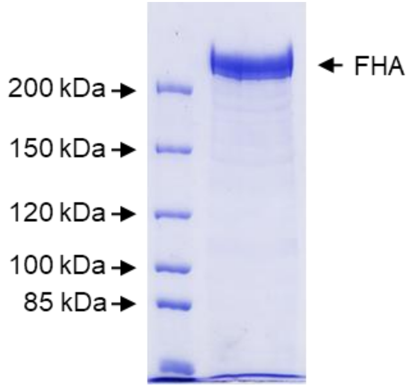

Figure S1. Coomassie-stained 5% SDS-PAGE of purified FHA. Mature FHA was purified by affinity chromatography on a Cellufine sulfate gel slurry from the supernatant of a *B. pertussis* culture as described in the Materials and Methods section.

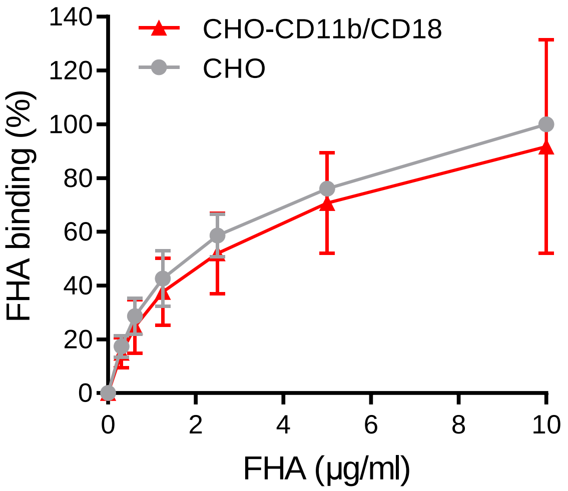

Figure S2. Mature FHA purified from cultures of *B. pertussis* does not recognize the CD11b/CD18 integrin. CHO and CHO-CD11b/CD18 cells (1×10^5) were incubated with different concentrations of purified FHA for 30 min at 4 °C, which was subsequently stained with an anti-FHA mAb and a secondary antibody conjugated with AF488. After analysis of FHA binding by flow cytometry, binding data were deduced from the MFI values and expressed as a percentage of FHA binding to CHO cells at a concentration of 10 µg/mL (taken as 100%). Each point represents the mean value ± SD of three independent experiments. No significant differences were observed among the binding curves of FHA to CHO and CHO-CD11b/CD18 cells ($p > 0.05$; ANOVA).

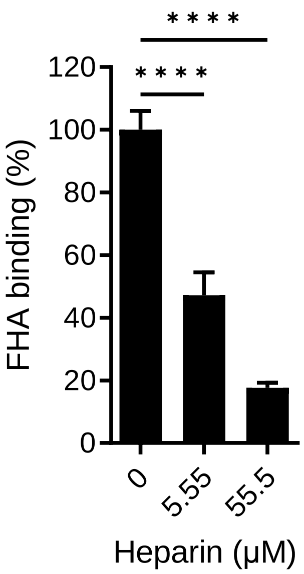

Figure S3. Heparin inhibits binding of FHA to CHO cells. Mock-transfected CHO cells (2×10^5) not expressing β_2 integrins were incubated with 5 µg/mL of purified FHA-Dy647 for 30 min at 4 °C in the presence of different concentrations of heparin (0, 5.55 and 55.5 µM) and analyzed by flow cytometry. Binding data were deduced from the MFI values and expressed as a percentage of FHA binding to cells in the absence of heparin (taken as 100%). Each bar represents the mean value with SD of three independent experiments performed in duplicate (****, $p < 0.0001$; ANOVA).
